# Characteristics and Predictors of Heavy Episodic Drinking (HED) among Young People Aged 16–25: The International Alcohol Control Study (IAC), Tshwane, South Africa

**DOI:** 10.3390/ijerph17103537

**Published:** 2020-05-19

**Authors:** Nadine Harker, Mukhethwa Londani, Neo Morojele, Petal Petersen Williams, Charles DH Parry

**Affiliations:** 1 Alcohol, Tobacco and Other Drug Research Unit, South African Medical Research Council, Cape Town 7505, South Africa; mukhethwa.londani@mrc.ac.za (M.L.); neo.morojele@mrc.ac.za (N.M.); Petal.petersen@mrc.ac.za (P.P.W.); cparry@mrc.ac.za (C.D.P.); 2 School of Family Medicine and Public Health, University of Cape Town, Cape Town 7505, South Africa; 3 School of Public Health, University of the Witwatersrand, Johannesburg 2050, South Africa; 4 Department of Psychiatry and Mental Health, University of Cape Town, Cape Town 7505, South Africa; 5 Department of Psychiatry, Stellenbosch University, Cape Town 7505, South Africa

**Keywords:** Alcohol, South Africa, drinking locations, young people, heavy episodic drinkers (HED)

## Abstract

In South Africa, little is known about alcohol consumption patterns, such as drinks consumed, container size, salience of alcohol price, affordability and availability, and perceptions of alcohol policies as potential predictors of heavy episodic alcohol (HED) use among young people. This paper examines predictors of HED among young people with specific consideration given to these alcohol consumption patterns. This study conducted in the Tshwane Metropole in 2014 employed multi-stage stratified cluster random sampling. Participants were between the ages 16–25 years. A structured questionnaire was used to collect data. Of the 287 (n = 678) participants who had used alcohol in the past six months and for whom we had complete consumption data, almost half were identified as heavy episodic drinkers (HEDs) and were significantly more likely to consume alcohol on a daily basis (*p* = 0.001). Having nightclub as the primary drinking location (*p* = 0.023) and drinking from a container size bigger than one standard drink (*p* = 0.014) were significant predictors for HED. HEDs were also more likely to have a perception that most people consume alcohol (*p* = 0.047). The results point to HED of alcohol among young people who drink in South Africa, highlighting the need for multicomponent interventions.

## 1. Introduction 

South Africa is one of the countries in sub-Saharan Africa with the highest levels of heavy episodic drinking (HED) [1]. Recently, it was proposed that, while only 30% of the population in South Africa drink alcohol, increases in consumption among young people are to be expected, given young people’s increased access to alcohol beverages, increased affordability, and aggressive marketing targeting this group [2]. Over the last decade, manufacturers of beer have all substantially increased their manufacturing capacity in Africa [3]. This is of great concern since alcohol consumption has already been identified as the leading risk factor for death and disability in sub-Saharan Africa and as the leading risk factor for disability-adjusted life-years lost (DALYs) among African male adolescents aged 15–24 years [2].

Research on adolescent alcohol consumption has shown that, from mid-adolescence to early adulthood, there are often increases in the amount and frequency of alcohol consumed, especially (heavy) binge drinking, which results in an increased risk for developing alcohol-related problems in later years [4,5]. Data from the 2016 South African National Demographic and Health Survey found that at least one in every four young person had consumed alcohol by the ages of 15–19, and that the percentage rises sharply by the ages of 20–35 [6].

This presents a major concern, since young people have greater vulnerability to alcohol [7]. Brain development takes place until around 25 years of age, and alcohol can lead to structural changes in the part of the brain involved in the learning process and, at higher levels, can permanently impair brain development [8]. Alcohol consumption also causes neurochemical modifications, with damage in memory, learning, and impulse control [9]. Furthermore, harmful use of alcohol has also been associated to unintentional and intentional injuries, crime, violence, and other social and health problems including sexual risk behaviours [10,11,12].

There is still a general lack of information about the harmful use of alcohol among young people in South Africa [2]. To our knowledge, there is no local data on whether alcohol consumption patterns, such as type of drink consumed, container size, salience of alcohol price, affordability/availability, or perceptions of alcohol policies play a role in predicting or mediating alcohol use among young people. Having up-to-date estimates on the nature of alcohol use among young people allows researchers to unpack possible predictors or drinking motives and to understand the antecedents and etiology of drinking behavior in this vulnerable population. It is also essential for informing the introduction of appropriate alcohol policy measures. 

This paper therefore aims to examine HED among young people aged 16–25 years in the Tshwane Metropole. We hypothesize that alcohol consumption patterns such as frequency of drinking, purchasing and location of drinking alcohol, degree of life satisfaction, the affordability and availability of alcohol, as well as spending practices and perceptions of policies regulating consumption serve as predictors of HED in low to middle income country settings. While important, the views of adolescents and young adults regarding alcohol adverts and their exposure to alcohol marketing in general were not the focus of this manuscript and are the subject of another sub-study coming out of the South African International Alcohol Control (IAC) study Morojele et al. (in press) [13].

## 2. Methods and Materials

### 2.1. Design and Sampling 

The cross-sectional study was conducted in 2014 in the City of Tshwane, South Africa. Tshwane is located within the province of Gauteng and overlaps into part of the northwest province. It is one of the six largest metropolitan municipalities in the country and the second largest in Gauteng, as measured by gross domestic product (GDP). Tshwane comprises a population of 3.275 million people [14].

The cross-sectional study is part of the South African arm of the International Alcohol Control (IAC) study [15]. The International Alcohol Control (IAC) Study is a multicountry collaborative project that aims to evaluate the impact of alcohol control policy across countries. South Africa is one of the countries, alongside Australia, England, Scotland, New Zealand, St Kitts and Nevis, Thailand, South Africa, Peru, Mongolia, and Vietnam, which are also participating in the cross-country study. The study was modelled on the International Tobacco Control study, and one of the key objectives is assessing the impact of alcohol policies in different cultural and socioeconomic contexts on policy-related behaviours [15]. 

The larger study targeted 3000 participants between the ages 16 and 65 years who had consumed alcohol in the past six months. The study had a response rate of 78%. We sought to have an adult sample of drinkers (2000 adults aged 18–65 years). The adolescent sample comprised drinkers and nondrinkers (1000 adolescents aged 16 and 17 years). Six hundred and seventy-eight participants, between the ages 16–25, reported using alcohol in the past 6 months; only 287 participants had complete alcohol consumption data (e.g., volume consumed and frequency of drinking in all 16 locations) and were therefore included in the analysis. We used the age range 16–25 for two main reasons: firstly, the United Nations use 15–24 years to define youth, and our paper was particularly focused on HED among youth. Secondly, this decision was also guided by data from our South African Community Epidemiology Network on Drug Use (SACENDU) that consistently reports that most persons admitted to treatment centres for specialist substance use treatment are <25, suggesting that this age group is particularly at risk.

A multi-stage stratified cluster random sampling design was employed. This involved the random selection of communities, census enumeration areas (EAs), and then households (“adult households” and “adolescent households”) within EAs. One adolescent and one adult were selected from each identified “adolescent household” and “adult household”. The margin of error which expresses the amount of random sampling error in the results (with finite population correction) was ±1.836%. The present paper only focuses on the baseline data for drinkers between the ages 16 and 25. 

### 2.2. Measures

A structured questionnaire which was adapted from the standard IAC questionnaire and translated into seTswana and Afrikaans [16] was used. 

#### 2.2.1. Demographic Measures

Data on age, gender, educational status, marital status, and race (ethnicity) were recorded. The “race” category refers to the demographic markers “White”, “Black”, and “Coloured”. The term “Coloured” refers to people of mixed ancestry in South African. Their continued use in South Africa is important for monitoring improvements in health and socioeconomic disparities (remnants of the old “apartheid” system), for identifying vulnerable sections of the population, and for planning effective prevention and intervention programmes. 

#### 2.2.2. Alcohol Consumption

Heavy episodic drinking (HED) was defined as consuming 8 standard drinks of 15 mL (12 g) of absolute alcohol for males and 6 standard drinks of 15 mL (12 g) of absolute alcohol for females on one occasion at any location at least monthly [16,17]. The alcohol consumption section of the questionnaire asked quantity and frequency for typical alcohol consumption at 16 locations (i.e., at your home; at someone else’s home; at nightclubs; at sports clubs; at restaurants, cafés/coffee shops; at theatres/movies; at workplaces; on plane trips; in motor vehicles; at sports events; at outdoor public places; at shebeens; at bars/pubs or taverns; at hotels; and at special events) over the past 6 months. From this, the quantity of absolute alcohol consumed was calculated as (number of containers) × (container size) × (percent absolute alcohol) for each of 13 beverage types (i.e., beer, low alcohol beer, and home brew beer; stout; wine; spirits; mixed cocktails; liqueurs; shooters; sherry, port, or vermouth; ciders; alcopops; and other) across the 16 locations. To determine average consumption of absolute alcohol (grams) by location, the amount of absolute alcohol consumed was summed for all beverage types consumed at each location.

Due to the small sample size, the frequency or pattern of drinking variable was recoded and trichotomized into “daily or almost daily drinking”, which included those participants reporting daily use plus those having a drink once in 2 days; “weekly use”, which indicates those who have a drink once in 3 to 7 days; and “less than weekly”, for those participants consuming alcohol less than weekly.

Given the problem of missing data on frequency and volume of alcohol consumed in all 16 drinking locations, primary drink variables were coded into “beer”, “wine”, “spirits”, “ciders”, and “other drinks” (which includes low alcohol beer and home brew beer; stout, liqueurs; shooters; sherry, port, or vermouth; and alcopops). Therefore, while 678 participants reported using alcohol in the past 6 months, only 287 participants had complete alcohol consumption data. 

#### 2.2.3. Socioeconomic Status (SES)

To determine socioeconomic status, assets at the household level were assessed. The asset index score was based on ownership of a radio, television, landline telephone, refrigerator, computer/laptop, and washing machine as well as access to electricity. Households with 1–4 assets were categorized as being on a “low SES”, 5–8 assets were categorized as “middle SES”, and 9 or 10 were categorized as “high SES”. This measure of socioeconomic status has previously been used in South Africa [18]. 

#### 2.2.4. Primary Drinking Location

The alcohol consumption section of the questionnaire asked questions relating to alcohol consumption at any of 16 locations (as described above) over the past 6 months. Due to low participant response on some of the location variables, data were recoded into alcohol consumption over the past 6 months “at your home”, “someone else’s home”, “nightclub”, “outdoors”, and “pub”, and the remaining locations were coded as “other locations”. 

#### 2.2.5. Purchasing Practices

For on- and off-premise purchase and prices, data were coded into the average amount of money in South African Rands (ZAR) spent on an alcohol beverage. 

##### RAPS4

We adapted the RAPS4 (four-item instrument) to ask about alcohol use in the past six months instead of the usual past year. The decision to use the RAPS4 measure was because it has been shown to be effective in detecting alcohol dependence in the past 12 months and would therefore be a measure of dependency whilst heavy episodic drinking may not necessarily indicate dependency but a pattern of drinking that could result in dependency or developing a substance-related disorder. In a recent publication, it was found that transition from use to abuse and dependency was longer for alcohol when compared to other drugs [19]. The RAPS4 has previously been used in one emergency room study in South Africa [20], but no previous studies on its psychometric properties among adolescents have been conducted. The four items with yes/no responses for symptoms of alcohol problems were combined into one dichotomous variable coded as the “presence of symptoms” (anyone scoring one or more symptoms) versus “no symptoms” (those with no symptoms, scoring 0).

##### Perceptions Relating to Being Served Alcohol or Being Caught When Intoxicated and Perceptions on the Proportions of Persons Consuming Alcohol

Perceptions on the likelihood of an intoxicated person being served alcohol at a specific location in the past 6 months was dichotomized into “not at all likely” or “very likely”. Perceptions on liquor bans included two questions: “the likelihood of being caught by police when drinking in a liquor-ban area” and “the likelihood of a drunk driver being caught by the police”. Perceptions regarding the proportion of people drinking alcohol were also included. 

##### Alcohol Availability, Affordability, and Price Influences

The survey also elicited participant views on whether they thought alcohol was currently available and affordable. Perceptions on price influence on the types and quantity of alcohol purchased were also collected. 

##### Primary Container Size

This study defined primary container size as the usual container size of the primary beverage at the primary drinking location. Container size was categorized by volume of absolute alcohol into “one standard drink”, “<one standard drink”, or “>one standard drink”. 

##### Life Satisfaction

Participants were asked how satisfied they felt with their life, ranging from zero being “completely dissatisfied” to 10 meaning “completely satisfied”. 

##### Policy Support

The policy support section of the survey measured participant’s views on 12 policy control measures on a 5-point Likert scale ranging from “strongly support” (1) to “strongly oppose” (5) with “don’t know”’ and “refuse” options. 

## 3. Procedures

The participants were recruited and interviewed in their homes by trained field workers. The questionnaires were loaded onto tablets. Interviews were only conducted after the interviewers received informed consent from the participants. The participants were each given a referral resource card and an incentive voucher worth ZAR30 (approximately Euro 1.70). The research was approved by the Research Ethics Committee of the South African Medical Research Council (EC 032-10/2012). 

### Statistical Analysis

Quantitative data analysis software, Stata version, 14.0 (StataCorp LP., College Station, TX, USA). Data were weighted by a statistician based at the South African Medical Research Council to factor in the complex sampling design used for this study. The sampling weights considered the survey design: the oversampling of non-Black participants at stage 1 and the number of eligible adults in the household at stage 4. Response rates were also calculated at the ward level, and the final weight was the product of the proportional and realization weights. Post hoc stratification weighting was therefore applied to have the approximate census distribution in the sum of the weights across the 16 strata plus the total weight approximately equal to the census population of the Tshwane study area. Finite sampling correction information for each stage was setup for the survey design to improve precision. 

First, logistic regression was conducted to detect associations by demographic variables, alcohol consumption characteristics, policy perceptions, and problematic alcohol use and treatment history between heavy episodic drinkers and non-heavy drinkers. Logistic regression as an analysis tool was chosen given that the aim of the study was to predict the outcome variable, heavy episodic drinking. A multiple logistic regression, with heavy episodic drinking as the dependent variable, was then used to determine if variables emerging as significant in the univariate logistic regression predicted heavy drinking. The multivariate analysis only included variables with significant relationships with the outcome variable and key demographic variables in the univariate logistic regression analysis. The analysis controlled for the following demographic variables, gender, race, age, education, income, and SES. 

Missing data occurred largely because of problems that initially occurred with the complicated programming of the software used in the tablets. Primarily, this has resulted in us excluding participants who did not have complete drinking data (quantity of alcohol consumed and/or frequency of drinking) over all 16 drinking locations. For a participant to remain in the analyses, they needed quantity of alcohol consumed and information on frequency of drinking over all 16 drinking location to be kept in. Therefore, while 678 participants, between the ages 16–25, reported using alcohol in the past 6 months, only 287 participants had complete alcohol consumption data, for example drinking in all 16 locations. The remaining participants did not report enough consumption data to calculate heavy episodic drinking status since they had frequency data for a given location but no consumption data for that location or vice versa, and therefore, we excluded these cases from the analyses. We conducted analysis to determine whether there were significant differences between those with missing consumption data and those with complete consumption data. With the exception of educational status (F = 1.50; *p* = 0.005), there were no other significant differences in terms of who was excluded.

## 4. Results

### 4.1. Demographic Profile of Participants and Proportion of Heavy Episodic Drinkers

Of the 678 participants who reported having used alcohol in the past 6 months, only 287 participants aged 16 to 25 years had complete alcohol consumption data for all locations. Of those for whom complete drinking data were available (n = 287), half (50.1%, 95% CI: 41.14–58.68) were identified as heavy episodic drinkers (HEDs). The mean age of the total sample was 21.2 years (SD = 0.22). Thirty-three percent were females, and 67% were males. Consistent with the demographics of the Tshwane metropolitan municipality, a large proportion of participants were of Black African descent (85%) (Table 1), with the majority being single (87.6%). Seventy-eight percent reported having only a primary school education. Additionally, 78.3% fell within the low-income category and 57.8% were of middle socioeconomic status. In this sample, 9.3% reported daily drinking. Of those, 16.5% reported heavy daily episodic drinking.

### 4.2. Associations between Heavy Episodic Drinking and Demographics, Alcohol Consumption, Perceptions of Being Served Alcohol, Being Caught When Intoxicated, and General Perceptions

Except for race (*p* = 0.021), there were no significant associations between HED and other demographic variables (Table 2). On the drinking location variable, heavy episodic drinkers were ten times more likely to have a nightclub as their primary drinking location compared to non-heavy drinkers (*p* = 0.013). HEDs were also less likely to have outdoor locations as their primary drinking location (*p* = 0.011) and were also 10% less likely to be weekly users of alcohol (*p* = 0.008). 

Heavy episodic drinkers were significantly less likely to have ciders or “other drinks” as their primary drink (*p* = 0.019 and *p* = 0.008, respectively). Regarding container size, heavy episodic drinkers were five times more likely to consume alcohol contained in an above average sized container and were more likely to spend between ZAR100–ZAR200 (1USD = ZAR19.04) at an on-premise location (*p* = 0.001 and *p* = 0.022, respectively). HEDs were also twice more likely (*p* = 0.048) to have the perception that intoxicated individuals were very likely to be served alcohol at a bar in the past 6 months. 

No significant results were found between HED and perceptions regarding alcohol availability and affordability. However, when compared to non-heavy drinkers, HED were less likely to indicate their support for earlier closing times for bars/nightclubs (*p* = 0.014). They were also less likely to support an increase in the price of alcohol (*p* = 0.012) and were less likely to support for an increase in alcohol taxes to government to cover costs for the treatment of alcohol use disorders (*p* = 0.027). Results for perceptions on implementing restrictions on alcohol advertising and restrictions on lowering breath alcohol limit did not reach significance (*p* = 0.08 and *p* = 0.057, respectively). No statistically significant difference was found between young people who drank heavily and those who did not in terms of symptoms of alcohol problems (RAPS4), life satisfaction, and treatment seeking. 

### 4.3. Predictors of Heavy Episodic Drinking of Alcohol among 16–25-year-olds (Multivariate Analysis)

In Table 3, the results indicated that being of “Coloured” descent (a neutral description in South Africa classifying people of mixed race/blended ancestry but does not include Asians) increased a person’s odds of being an HED (AOR 4.93; *p* = 0.017). Having nightclub as the primary drinking location increased a person’s odds of being a HED, and drinking from a container size bigger than one standard drink (*p* = 0.014) was significant predictors for heavy episodic drinking. In contrast, drinking less than weekly decreased a person’s odds of heavy episodic drinking with daily drinking. Heavy episodic drinkers were less likely to think that only a few people drink alcohol (*p* = 0.047).

## 5. Discussion 

The study found that, of those young people aged 16 to 25 for whom we had complete drinking consumption data (n = 287), close to 50% reported “heavy episodic drinking”. If we were to consider the total community sample including those for whom consumption and drinking location data were missing (n = 687), 21% indicated HED. This resonates with other South African data reporting heavy consumption of alcohol. Data from the South African Youth Risk Behaviour Survey found that, nationally, 25.1% (23.3–27.0) of Grade 8–11 learners had drank five or more drinks of alcohol within a few hours on one or more days in the month preceding the survey [21]. Additionally, data from the World Health Organisation (WHO) found that adult drinkers on average drank 27.1 L of pure alcohol, recorded as the highest in Africa [1]. 

Our study also found that participants who met criteria for heavy episodic drinking reported consuming alcohol on a daily basis. These results are of great concern and suggests that young people who drank daily drank at least eight standard drinks of 15 mL (12 g) of absolute alcohol (men) or six standard drinks of 15 mL (12 g) of absolute alcohol (women) on one occasion; this is higher than the cutoffs reported in other surveys. This pattern of drinking found in this study exposes adolescents and young people to a broad spectrum of risks [22] including the risk of developing a substance-related disorder. Whilst the RAPS4 measurement which is a substance-related disorder-screening tool for dependency did not reach significance in this study, the lack of significance can be explained by the findings from a recent publication confirming that the median age of onset of use was around 20 years and that of regular use was at 21 years. The study further found that transitioning from regular alcohol use to alcohol abuse and from abuse to alcohol dependence occurs later in life, generally in the late 40s, and takes people an average of 5 years to transition from regular use to abuse and 7 years to transition from use to dependence [19]. Considering the length of time it takes people to transition to more harmful levels of alcohol use, there is an opportunity to identify regular users of alcohol via screening and to provide them with brief interventions to help them reduce their risk of progressing to abuse or dependence [19]. However, careful consideration should be given to evidence for effectiveness of specific interventions [23]. Of note are random breath testing, graduated licensing, and the enforcement of drunk driving laws and minimum age restrictions [22]. Early interventions and timely screening for heavy episodic drinking in multiple settings is also recommended. 

Participants from predominantly Coloured descent were more likely to be heavy episodic drinkers when compared to Black African or White participants in the study. In other studies, it has been hypothesized that greater access to income; socioeconomic disadvantage; and other contextual factors such as drug marketing practices and cultural differences between Black African, White, and Coloured communities may account for differences in substance use behavior between different ethnic groups in South Africa [24]. On the other hand, this finding may also speak to continuing legacies from the past. During the “apartheid” era, alcohol was used to economically and socially control mine and farm workers through a practice called the “dop” or “tot” system [25]. The “dop” system is the payment of workers with alcohol in lieu of wages. While the dop system had its origins in the early years of colonial settlement in the Cape Colony (where the Coloured population largely resided), the practice became an institutionalized element of farming and mining practice in the Cape (and other parts of South Africa) over three centuries [26]. This system of paying farm workers with alcohol had a specifically profound effect on “Coloured” communities and resulted in a myriad of poor social indicators for farm workers [26]. Furthermore, it also deeply influenced this community’s self-perception and sense of identity [27], and perhaps its damaging imprint still remains and should not be rejected as a plausible theory for heavy episodic drinking in this vulnerable population. Regardless of the reasons for these differences, our findings highlight a vulnerability in this population and the need for interventions targeting heavy episodic alcohol use. Our findings suggest that such interventions should focus on reducing heavy episodic drinking and further on investigating factors that might be putting certain drinkers in Tshwane at greater risk for heavy drinking. 

Furthermore, this study found that nightclubs are the primary drinking location for persons in this age category and a subsequent predictor for HED. This is in keeping with other literature sources which have reported that a substantial proportion of at-risk drinking takes place in nightlife environments such as bars, pubs, and nightclubs [28]. A study conducted among young Swiss men, for example, found that drinking volumes at own home, friends’ homes, bars/pubs/taverns, discos/nightclubs, outdoor public places, and special events were consistently significantly associated with DSM-V alcohol use disorder criteria [29]. Additionally, data emerging from a study on alcohol use and sexual behaviour among risky drinkers and bar/shebeen patrons in the Gauteng province, South Africa reported high levels of alcohol consumption among patrons [30]. This is of concern since the high levels of alcohol consumption in nightlife environments carry numerous dangers such as increased risk for traffic accidents, violence [27], excessive drunkenness, antisocial behavior [30], as well as the risk for contracting an infectious disease such as HIV/AIDS [30]. While a range of interventions such as cooperative approaches that seek to train staff in licensed premises and to engage nightlife industries in socially responsible operations have been recommended, a recent systematic review found that the clearest indication of effectiveness comes from multicomponent programmes [31]. These programmes combine community mobilization, responsible beverage service (RBS) training, house policies, and stricter enforcement of licensing laws [31,32]. 

We also found that container size was a predictor of heavy episodic drinking. Recent studies have found that the size of glasses or container in which alcohol is purchased can affect consumption [33]. In South Africa, youth are targeted by the alcohol industry that is determined to explore a previously untapped market and youth are therefore vulnerable to youth-specific marketing strategies. Given the risk associated with drinking alcohol in containers with alcohol content greater than roughly one standard drink, this is something that should be considered in South Africa or countries with similar cultures of heavy episodic drinking [34]. 

Whilst the logistic regression analysis found associations between heavy episodic drinking and policy support, results from the multiple regression showed no significant differences except on perceptions of drinking behaviours in the general population. In fact, heavy episodic drinkers were of the opinion that “most” rather than a “few” people drink. Interestingly, the social norms theory suggests that perceptions and beliefs of what is “normal” behaviour by others will influence one’s own behavior [35], and therefore, the amount a person drinks can be influenced by their perception of drinking by others [36]. 

While the study provided useful insights into harmful alcohol use among young people, the study has limitations, foremost of which being that the research was cross-sectional which makes it difficult to make inferences of causality. Secondly, the data are also specific to the Tshwane Metropole, and it is unknown whether the findings would generalize to other parts of South Africa, particularly rural areas. Thirdly, the study was plagued by missing consumption variables as a result of complications with the data collection software. This resulted in a low level of usable data and therefore a smaller than expected sample size; we had to dichotomize most of the study variables for analysis purposes, and this might mask certain associations with HED behaviours. In addition, the complexity and length of the questionnaire which contained numerous skip patterns and loops may also have posed a problem. Future research should ensure that questionnaires are kept short and concise. Added to this is that self-report questionnaires are subject to response bias and have their limitations and especially about typical quantities of alcohol consumed. All of the above contributed to the low sample size, and therefore, the study is limited in its inability to draw concise conclusions and should therefore be interpreted with caution. However, it also paves the way and justifies the need for further research that investigates the risk profiles of young people. 

## 6. Conclusions

This paper explored HED among young people in Tshwane, further examining predictors of HED in this select population. More than half of the study sample was identified as heavy episodic drinkers. This is of concern given the associated burden alcohol places on the health and welfare services and larger society, highlighting the need for multicomponent interventions that target specifically young people. Given that nightclubs emerging as primary drinking locations, that certain populations appearing more vulnerable and at risk for HED, and that drinking from a container size bigger than one standard drink were significant predictors for HED, consideration should be given to early interventions and timely screening for heavy episodic drinking in multiple settings. Some multi-pronged interventions may require amendments to current policy as well as stricter enforcement of alcohol policy laws. 

## Figures and Tables

**Table 1:** Survey participant demographic details (n = 287).

Demographic and Drinking Variables	Combined Dataset		Non-Heavy Drinkers		Heavy Episodic Drinkers	
	n	%	n	%	n	%
**Gender**						
Male	178	67.0	87	62.4	91	72.2
Female	109	33.0	56	37.6	53	27.8
Total *	287	100	143	100	144	100
**Age in Years**						
16–17	32	11.0	23	15.0	9	6.5
18–19	52	19.2	29	19.2	23	19.2
20–25	203	69.8	91	65.9	112	74.3
Total *	287	100	143	100	144	100
**Race**						
Black African	211	84.6	99	82.1	112	87.5
Coloured	47	6.4	23	4.2	24	8.8
White	24	9.0	17	13.7	7	3.8
Total *	282	100	139	100	143	100
Income *						
Low	140	78.3	65	75.9	75	80.7
Middle/High	26	21.7	10	24.1	16	19.3
Total *	166	100	75	100	91	100
Education *						
Primary	12	2.8	3	0.5	9	5.5
Secondary	243	84.8	128	92.6	115	75.7
High	32	12.4	12	6.9	20	18.8
Total *	287	100	143	100	144	100
**Marital Status**						
Never married	183	89.2	80	85.8	103	92.5
Marital status other	26	10.9	14	14.2	12	7.5
Total *	209	100	94	100	115	100
**Socioeconomic Status (SES)**						
Low SES	49	14.6	17	15.1	32	14.0
Middle SES	163	57.8	80	53.4	83	62.8
High SES	75	27.6	46	31.5	29	23.2
Total *	287	100	143	100	144	100
**Drinking pattern**						
Daily drinking	31	9.3	4	3.0	27	16.5
Weekly use	86	27.6	23	17.6	63	39.2
Less than weekly	170	63.1	116	79.4	54	44.3
Total *	287	100	143	100	144	100

* Missing data.

**Table 2:** Univariate logistic regression.

Independent Variables	OR	95% CI	*p*-Value
**Gender**			
Male	1.00		
Female	0.639	0.270–1.512	0.286
Race			
African	1.00		
White	0.258	0.099–0.675	**0.009**
Coloured	1.967	0.903–4.284	0.084
Age			
16–17	1.00		
18–19	2.316	0.503–10.668	0.261
20–25	2.611	0.485–14.046	0.244
Marital Status			
Never married	1.00		
Marital status other	0.486	0.218–1.084	0.074
Personal Income			
Low	1.00		
Middle/High	0.754	0.191–2.972	0.667
Education			
Primary	1.00		
Secondary	0.079	0.014–0.446	**0.007**
Tertiary	0.265	0.029–2.421	0.221
Socioeconomic Status (SES)			
Low SES	1.00		
Middle SES	0.533	0.176–1.619	0.248
High SES	0.477	0.127–1.790	0.252
Primary Drinking Location			
Home	1.00		
Someone Else’s Home	2.548	0.892–7.281	0.077
Nightclub	5.396	1.570–18.541	**0.011**
Outdoors	0.219	0.057–0.841	**0.029**
Pub	2.604	0.881–7.692	0.080
Other locations	2.057	0.840–5.036	0.107
Drinking Pattern			
Daily use	1.00		
Weekly use	0.398	0.075–2.116	0.260
Less than weekly	0.100	0.020–0.503	**0.008**
Consumption Variables			
Primary Drink			
Beer	1.00		
Wine	0.620	0.173–2.227	0.440
Spirits	0.320	0.089–1.152	0.078
Cider	0.357	0.155–0.822	**0.019**
Other drinks	0.124	0.029–0.535	**0.008**
Container size			
Below Average	0.139	0.014–1.373	0.086
Average	1.00		
Above Average	5.224	2.212–12.339	**0.001**
Purchasing practices—on-premise typical spending			
R0–R99	1.00		
R100–R199	8.013	1.412–45.484	**0.022**
R200+	4.235	0.454–39.474	0.187
Purchasing practices—off-premise typical spending			
R0–R249	1.00		
R250– R499	0.753	0.172–3.301	0.686
R500+	0.336	0.034–3.315	0.324
Rapid Alcohol Problems Screen 4 (RAPS4)			
0	1.00		
±1	1.725	0.806–3.689	0.148
Life Satisfaction			
Completely dissatisfied	1.00		
Completely satisfied	0.682	0.281–1.656	0.374
Perceptions on the Likelihood That a Drunk Person Be Served Alcohol at Specific Locations in the Past 6 Months			
Bar			
Not at all likely	1.00	-	-
Very likely	2.06	1.01–4.20	**0.048**
Nightclub			
Not at all likely	1.00	-	-
Very likely	1.57	0.89–2.77	0.113
Sports club			
Not at all likely	1.00	-	-
Very likely	2.14	0.99–4.66	0.054
Friend’s home			
Not at all likely	1.00	-	-
Very likely	1.95	0.94–4.03	0.070
Shebeen			
Not at all likely	1.00	-	-
Very likely	1.06	0.54–2.08	0.860
Perceptions on Liqour Bans			
Likelihood of being caught by the police when drinking in a liquor-ban area			
Not at all likely	1.00	-	-
Very likely	1.13	0.58–2.19	0.709
Likelihood of a drink driver caught by the police			
Not at all likely	1.00	-	-
Very likely	1.35	0.57–3.19	0.467
Alcohol Affordability and Availability			
How Affordable is alcohol to you currently?			
Unaffordable	1.00	-	-
Affordable	1.00	0.50–1.99	0.995
How Available is alcohol to you currently?			
Unavailable	1.00	-	-
Available	2.09	0.91–4.80	0.077
Does the price of alcohol influence the types of beverages purchased			
Not at all	1.00	-	-
A great deal	1.06	0.51–2.18	0.868
Does the price of alcohol influence the quantity of alcohol purchased			
Not at all	1.00	-	-
A great deal	1.00	0.44–2.31	0.994
Policy Support			
Support for a purchase age of 21 years			
Yes	1.00	-	-
No	1.10	0.55–2.19	0.781
Support for restrictions on numbers of outlets in the community			
Yes	1.00	-	-
No	0.75	0.31–1.82	0.505
Support for earlier closing times for bars/nightclubs			
Yes	1.00	-	-
No	0.42	0.21–0.82	**0.014**
Support for earlier closing times for hotels			
Yes	1.00	-	-
No	1.31	0.75–2.28	0.321
Support for earlier closing times for bottle stores and supermarkets			
Yes	1.00	-	-
No	0.53	0.25–1.13	0.095
Support for the increase in the price of alcohol			
Yes	1.00	-	-
No	0.41	0.21–0.80	**0.012**
Support for restrictions on alcohol marketing/advertising			
Yes	1.00	-	-
No	0.52	0.24–1.12	0.088
Support for restrictions on lowering breath alcohol limit			
Yes	1.00	-	-
No	1.85	0.98–3.50	0.057
Support for more random breath testing			
Yes	1.00	-	-
No	1.06	0.36–3.08	0.914
Support for an increase in alcohol taxes to pay for alcohol treatment			
Yes	1.00	-	-
No	0.46	0.23–0.90	**0.027**
Support for an increase in alcohol taxes to lower other taxes			
Yes	1.00	-	-
No	1.00	0.53–1.89	0.992
Support for an increase in alcohol taxes for government purposes			
Yes	1.00	-	-
No	0.72	0.37–1.40	0.304
Support for taxing drinkers to pay for the cost for harm to society			
Yes	1.00	-	-
No	0.70	0.38–1.29	0.232
Treatment			
In the past 6 months have you received help to reduce level of drinking			
Yes	1.00	-	-
No	2.21	0.53–9.19	0.255
What proportion of people drink alcohol?			
Most people drink	1.00	-	-
Some people drink	0.28	0.11–0.71	**0.010**
A few people drink	0.58	0.18–1.93	0.353

* significant *p*-values are reported in bold script.

**Table 3:** Multiple logistic regression and determinants of heavy episodic drinking among 16–25 year old persons.

Determinants of HED Title	AOR	95% CI	*p*-Value
Gender			
Male	1.00	-	-
Female	1.31	0.40–4.33	0.639
Race			
African	1.00	-	-
White	0.85	0.06–11.12	0.891
Coloured	4.93	1.40–17.44	**0.017**
Age Distribution			
16–17	1.00	-	-
18–19	0.77	0.00–181.67	0.881
20–25	2.07	0.02–268.07	0.754
Marital Status			
Never married	1.00	-	-
Marital status other	0.30	0.06–1.48	0.129
Personal income			
Low	1.00	-	-
Middle/High	0.84	0.18–3.98	0.817
Primary drinking location			
Home	1.00	-	-
Someone else’s Home	1.32	0.37–4.63	0.647
Nightclub	6.91	1.35–35.26	**0.023**
Outdoors	0.02	0.00–0.33	**0.009**
Pub	1.53	0.38–6.06	0.525
Other locations	3.99	0.70–22.81	0.111
CONSUMPTION			
Primary Drink			
Beer	1.00	-	-
Wine	0.47	0.06–3.47	0.430
Spirits	0.07	0.01–0.31	**0.002**
Cider	0.42	0.13–1.31	0.124
Other drinks	0.12	0.02–0.82	**0.033**
DRINKING Pattern			
Daily drinking	1.00	-	-
Weekly use	0.20	0.02–2.25	0.175
Less than weekly	0.08	0.01–0.62	**0.019**
Container size			
Below Average	0.08	0.00–2.94	0.157
Average	1.00	-	-
Above Average	6.19	1.51–25.30	**0.014**
Drunk Person be served alcohol at the Bar in past 6 months			
Not at all likely	1.00	-	-
Very likely	1.22	0.42–3.51	0.696
POLICY SUPPORT			
Support for earlier closing times for bars/nightclubs.			
Yes	1.00	-	-
No	0.62	0.25–1.57	0.294
Support for restrictions—Alcohol marketing/advertising			
Yes	1.00	-	-
No	0.29	0.09–0.91	**0.036**
Support for the increase in alcohol taxes to lower other taxes			
Yes	1.00	-	-
No	1.04	0.45–2.42	0.927
What proportion of people drink alcohol?			
Most people drink	1.00	-	-
A few people drink	1.59	0.16–16.09	0.679
Some people drink	0.28	0.08–0.98	**0.047**

* significant *p*-values are reported in bold font.
